# Expression of hormonal receptors in osteosarcomas of the jaw bones: Clinico-pathological analysis of 21 case

**DOI:** 10.4317/medoral.18729

**Published:** 2013-10-13

**Authors:** Hugo R. Domínguez-Malagón, Esther González-Conde, Ana M. Cano-Valdez, Kuauhyama Luna-Ortiz, Adalberto Mosqueda-Taylor

**Affiliations:** 1 Departament of Pathology, Instituto Nacional de Cancerología, México; 2 Departament of Head and Neck Surgery, Instituto Nacional de Cancerología, México; 3 Health Care Department, Universidad Autónoma Metropolitana Xochimilco, México

## Abstract

Background: Sexual hormones have an important role in many hormone-dependant tumors like breast and prostate carcinomas, and also a relationship has been found with bone metabolism and bone tumors. Some studies have demonstrated that the expression of hormonal receptors (HR) in osteosarcomas (OS) of long bones is associated with gender, histological grade, histological type, and possibly may be connection with pathogenesis and evolution. However, to our knowledge there are no studies of HR in osteosarcomas of craniofacial bones (OS-CF). Objectives: To assess the expression of hormonal receptors in OS-CF. Material and Methods: Twenty one cases of OS-CF were included in this study. Clinical outcome was obtained from clinical charts. Histological sections were reviewed, and immunohistochemistry studies for estrogen, progesterone and androgen receptors were performed. Results: A striking female predominance was found (2:1), with a median age of 35 years. The predominant type of OS was osteoblastic (52.4%), and histological grade was high in 86%. Follow-up was obtained in 13 cases and ranged from 6 to 118 months (median 29 months). There were 8 patients (61.5%) dead or alive with progressive disease in the last follow up. Negative expression of HR was found in 19/21 cases; one showed weak nuclear expression for estrogen receptor, and another for androgen receptor. Progesterone receptor was negative in all cases. Conclusions: OS-CF mostly affected females, most of them were of the osteoblastic type and of high grade. Hormonal expression was practically negative in osteosarcoma of craniofacial bones.

** Key words:**Osteosarcoma, jaws, estrogen, progesterone, androgen receptors.

## Introduction

Participation of hormones in bone metabolism is a well known phenomenon, either in a direct or indirect form. Among the most frequently studied ones are: growth hormone, prolactin, somatomedin, growth factor similar to insulin, paratohormone, thyroid hormones, and steroid hormones. The last three have a role in maintenance of integrity of bone structure. The osteoblasts are able to produce, from circulating steroids, biologically functional estrogens and androgens (1). There are research works directed to clarify the possible participation of sex hormones in the pathogenesis of osteosarcoma (OS) of long bones (2) that show a predominant occurrence in adolescent males. It has been demonstrated that androgens promote osteoblastic differentiation (3), and androgen receptors (AR) have been identified in OS, and that they are more abundant in metastatic OS (4), which suggests that anti-androgenic treatment could be useful as a therapeutic modality. While AR have been found to be more numerous and to have higher affinity in patients previously treated with chemotherapy-radiotherapy (5), there are some studies that have not demonstrated a direct interaction of androgens and their receptors, suggesting that diverse pathways of action may exist in the pathogenesis of bone tumors (6).

Neoplastic cells of OS show a nuclear pattern of staining for AR and estrogen receptors (ER) (7), whereas reactive osteoclasts may show cytoplasmic staining for ER (8-11).

With the purpose of identifying possible prognostic factors and treatment guidelines, there have been some attempts to correlate expression of HR with several clinical and histological signs. The expression of AR has been correlated with worse prognosis and a shorter metastasis free period after treatment (12).

Other authors (13) have observed that the expression of ER was more intense with better tumor differentiation. On the other hand, Fohr et al. (14) studied the in-vitro effects of sexual steroids in cell lines of human OS, segregating groups according to histological grade and patient´s gender, and no significant differences were found. Most published works refer to the expression of HR in long bones, but no studies of craniofacial osteosarcomas (CF-OS) were found in the literature review. Craneofacial bones are embryologically distinct, as they evolve from intramembranous ossification, while long bones are formed by endochondral ossification.

The objective of the present work was to assess the expression of HR in CF-OS, and to compare the results with those found in studies of OS of the long bones published in the literature.

## Material and Methods

Twenty-one cases of CF-OS were collected (12 were from the pathology archives of the Instituto Nacional de Cancerología and 9 from the consultation files of the authors). Inclusion criteria were: cases that had confirmed diagnosis of osteosarcoma of the jawbones which have enough tissue to perform immunohistochemical analysis with three different hormonal receptors. Those cases that had not paraffin-embedded tissue were excluded, as well as those cases that were not useful for performing the immunohistochemical study due to technical difficulties on their process.

In 13 cases complete clinical information and follow-up was available. Clinical stage was evaluated with the pathologic findings of surgical resection using the TNM system of the American Joint Committee on Cancer 2002 (15). The histological slides were reviewed to standardize histological criteria.

Immunohistological studies were performed in archival paraffin embedded tissue using antibodies against estrogen receptor (ER) (1:400), progesterone receptor (PR) (1:100) and androgen receptor (AR) (1:100) (BIO SB Inc. Santa Bárbara CA), with the biotin-streptavidin-peroxidase method. The reaction was considered positive when the signal was exclusively nuclear, while the cytoplasmic reaction was regarded as negative.

## Results

The tumors studied in this series occurred with more frequency in the female gender (66.7% with a F:M ratio of 2:1). The median age of presentation was 35.2 years (range 16 to 68 years). The most frequent histological type observed was osteoblastic OS (11 cases, 52.4%), followed by fibroblastic (7 cases, 33.3%). The majority were classified as high-grade tumors (18 cases, 86%) ( Table 1) and almost half of the patients were in clinical stage IIA ( Table 2).

**Table 1:**
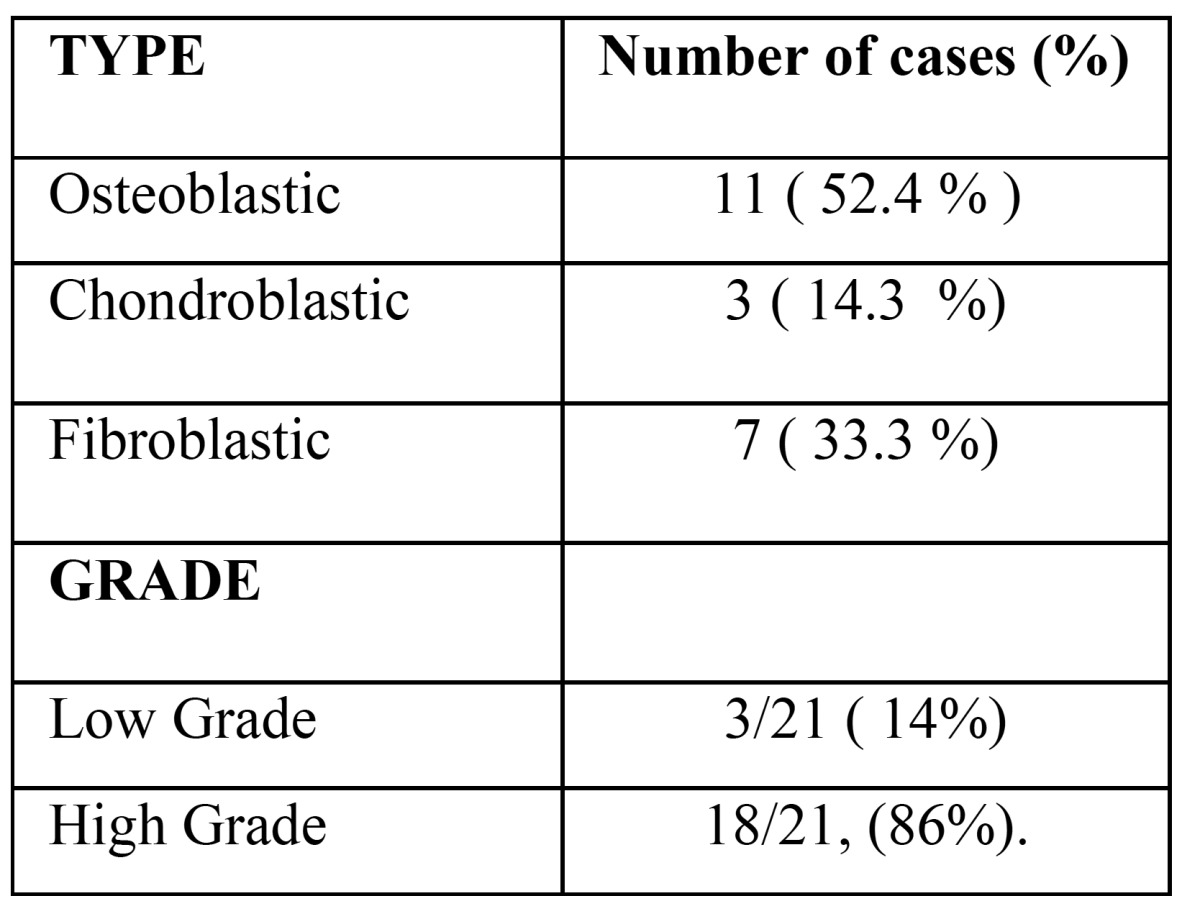
Histological features of osteogenic sarcoma in the present series (n=21).

**Table 2:**
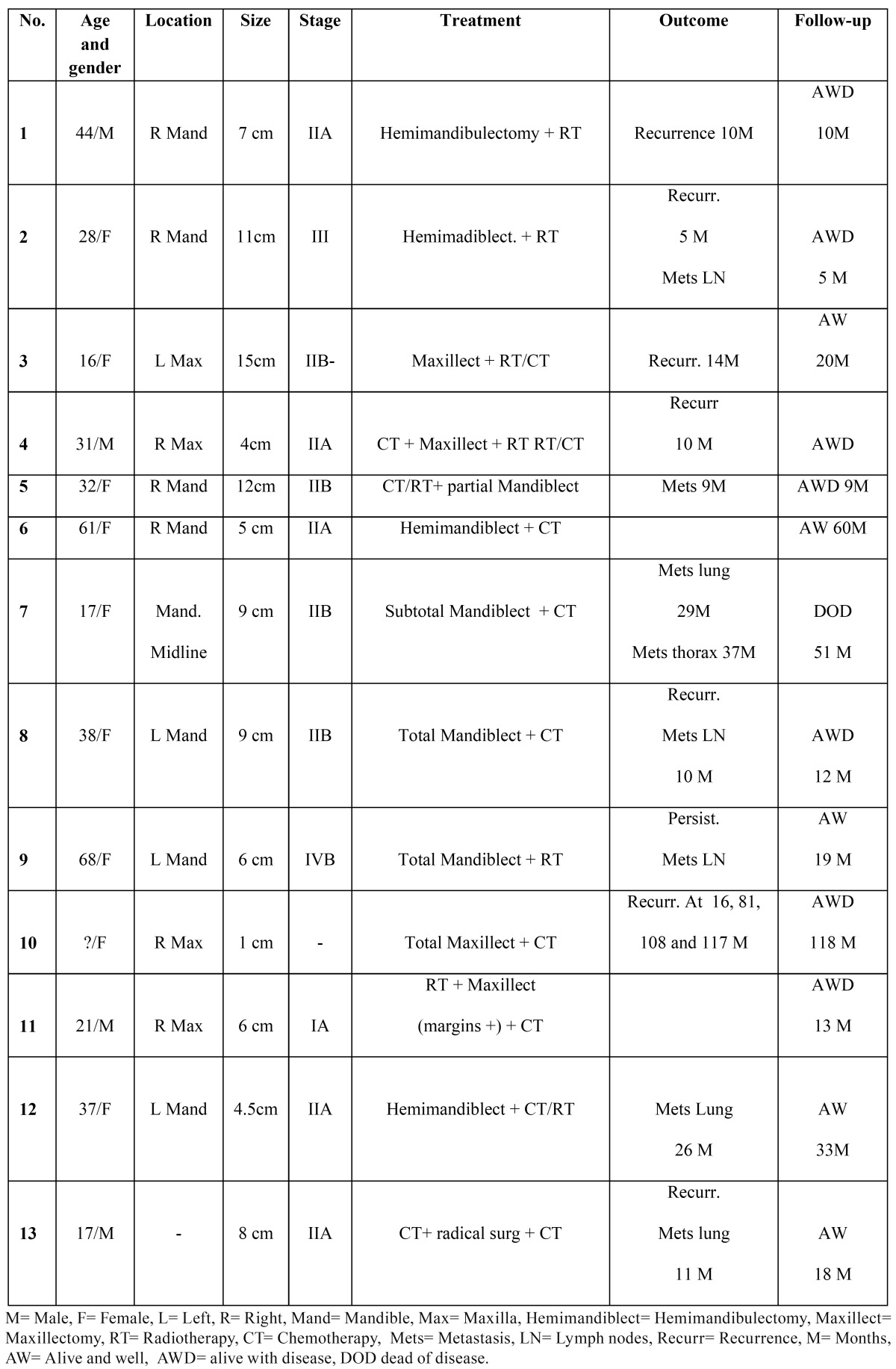
Clinical features of jaw osteosarcomas in the present series (n=13).

The immunohistochemical studies expressed the following: 19 cases (90.5%) were negative for HR, one case showed nuclear signal for ER, another case displayed weak nuclear signal for AR , and all cases were negative for PR.

Complete clinical information was obtained in 13 cases with follow-up ranging from 7 to 109 months (median: 30 months). Seven patients (53.8%) developed metastases, three in the cervical lymph nodes, two in the lung, one in the lung and thorax and one in an unspecified site. Rapid progression occurred in 4 patients (30%) and eight cases (61.5%) either died of disease or presented advanced disease in the last follow-up ( Table 2).

## Discussion

CF-OS are uncommon tumors, comprising 6-10% of all OS, and they affect preferentially the body of the mandible (16,17). The highest incidence is in the 3rd and 4th decades of life. In this study the median age of presentation was 32 years, which is similar to previous reports, although there were two patients under the age of 20 (16 and 17 years, respectively).

In the present series, a frank predominance in women was found with a F:M ratio of 2:1, while in the literature CF-OS is reported to be more frequent in males, although a slight female predominance has been reported in series from Mexico (18) and Japan (19). At the present time we can not speculate if this gender tendency is related to sociodemographic and racial factors or if it is due to intrinsic conditions of the neoplasia or the bone type.

Many series in the literature report that the chondroblastic variety is the most frequent histological type of CF-OS (17), which is in disagreement with the present work in which the predominant type was osteoblastic, but the cause of this difference is unknown. The most frequent consequence of treatment failure in CF-OS is local recurrence, which occur in up to 50%, and metastases in 30% within two years after resection of the primary tumor. The favorite metastatic site is lung (25%) followed by lymph nodes and central nervous system (20,21). Our recurrence rate was 30%, and metastases occurred in 38%.

There are series of CF-OS in the literature that report a great proportion of high-grade tumors (17-19), with frequencies ranging from 54% to 90%, similar to the 86% figure found in the present work.

At puberty, steroid sex hormones stimulate endochondral bone formation of long bones. OS of these bones is more frequently seen at this age, and the expression of HR has been well demonstrated; however, in the adult, bone maintenance is based mainly in androgen metabolism in men and also in women. In has been reported that in non-neoplastic tissue of long bones, the osteoblasts show nuclear expression of ER, while in osteoclasts it is cytoplasmic (8-10).

It is accepted that in the appendicular long bones the ossification is endochondral, i.e. from the cartilage at the bone ends. On the other hand, ossification of cranial flat bones is of membranous type, by direct differentiation of mesenchymal cells into osteoblasts; however, in some of craniofacial bones there are spots of endochondral ossification (22-27) and, depending on its origin axial or apendicular, bone development has complex molecular signaling pathways for its mineralization, involving tissular growth factors and hormones that play an important role (28); however, their precise actions in cranio-facial bone development is not completely understood, as the effect of sex hormones in endochondral mineralization has been explored only in the mandibular condyle (22), and to our knowledge, there are no reports on the action of steroid hormones in membranous bones.

In the present series there was absence of expression of HR in the majority of cases, as only in two of them weakly positive signals for AR and ER were detected respectively. Other two cases showed osteoclasts with cytoplasmic expression for HR.

We believe that the low expression of HR may be related to the type of bone studied, and perhaps the focal signal of HR is due to transitory endochondral mineralization in the craniofacial bones (29). Another explanation would be that as membranous ossification is originated in a pluripotential cell, the expresión of HR could be present but with a lesser intensity.

The expression of HR in long bones in relationship to differentiation has been previously studied; in this respect, Liu et al. (13) observed that the intensity of the reaction was in direct relationship with tumor differentiation.

In conclusion, our results indicate that HR have negative or minimal expression in CF-OS, which are in discrepancy with studies that have explored HR in OS of the long bones. The reason for this difference could be the differences in the mechanism of ossification and bone metabolism that occurs in jaws. Other differences with respect to most previous publications are the female predominance (2:1), and the osteoblastic predominance found in our series.

## References

[1] Saito H, Yanaihara T (1998). Steroid formation in osteoblast-like cells. J Int Med Res.

[2] Walker MJ, Chaudhuri PK, Beattie CW, Das Gupta TK (1980). Steroid receptors in malignant skeletal tumors. Cancer.

[3] Benz DJ, Haussler MR, Thomas MA, Speelman B, Komm BS (1991). High-affinity androgen binding and androgenic regulation of alpha 1(I)-procollagen and transforming growth factor-beta steady state messenger ribonucleic acid levels in human osteoblast-like osteosarcoma cells. Endocrinology.

[4] Kushlinskii NE, Mitin VN (1992). Androgen Receptors in the cytosol fraction of a spontaneous ostegenic sarcoma in dogs. Vopr Onkol.

[5] Kushlinskii NE, Siniukov PA, Petrovichev NN, Vasil'ev AV (1987). Cytoplasmic androgen receptors in malignant bone neoplasms and their relation to the morphological characteristics of the tumor. Arkh Patol.

[6] Bassalyk LS, Kushlinskii NE, Revazova ES, Degtiar VG (1988). Testosterone stimulation of the growth of a human osteogenic sarcoma strain transplanted into athymic rats. Eksp Onkol.

[7] Zhuang YH, Bläuer M, Pekki A, Tuohimaa P (1992). Subcellular location of androgen receptor in rat prostate, seminal vesicle and human osteosarcoma MG-63 cells. J Steroid Biochem Mol Biol.

[8] Vidal O, Kindblom LG, Ohlsson C (1999). Expresion and localization of estrogen receptor-beta in murine and human bone. J Bone Miner Res.

[9] Ikegami A, Inoue S, Hosoi T, Mizuno Y, Nakamura T, Ouchi Y (1993). Immunohistochemical detection and northern blot análisis of estrogen receptor in osteoblastic cells. J Bone Miner Res.

[10] Sutherland MK, Hui DU, Rao LG, Wylie JN, Murray TM (1996). Immunohistochemical localization of the estrogen receptor in human osteoblastic SaOS-2 cells: association of receptor levels with alkaline phosphatase activity. Bone.

[11] Colston KW, King RJ, Hayward J, Fraser DI, Horton MA, Stevenson JC (1989). Estrogen receptors and human bone cells: immunocytochemical studies. J Bone Miner Res.

[12] Bassalyk LS, Kushlinskii NE, Solov'ev IuN, Siniukov PA, Fedenko AN (1989). Steroid hormone receptors in osteogenic sarcomas. Vopr Onkol.

[13] Liu J, Le X, Li Y (1998). Pathological study of ER and PR in osteosarcoma. Zhonghua Wai Ke Za Zhi.

[14] Fohr B, Schulz A, Battmann A (2000). Sex steroids and bone metabolism: comparison of in vitro effects of 17beta-estradiol and testosterone on human osteosarcoma cells lines of various gender and differentiation. Exp Clin Endocrinol Diabetes.

[15] Greene FL, Page DL, Fleming ID, Fritz A, Balch CM, Haller DG (2002). AJCC Cancer Staging Manual. 6th ed..

[16] Dorfman HD, Czerniak B (1995). Bone cancers. Cancer.

[17] Clark JL, Unni KK, Dahlin DC, Devine KD (1983). Osteosarcoma of the jaw. Cancer.

[18] Delgado R, Maafs E, Alferain A, Mohar A, Barrera JL, Zinser J (1994). Osteosarcoma of the jaw. Head Neck.

[19] Tanzawa H, Uchiyama S, Sato K (1991). Statistical observation of osteosarcoma of the maxillofacial region in Japan. Analysis of 114 Japanese cases reported between 1930 and 1989. Oral Surg Oral Med Oral Pathol.

[20] Garrington GE, Scofield HH, Cornyn J, Hooker SP (1967). Osteosarcoma of the jaws. Analysis of 56 cases. Cancer.

[21] Bertoni F, Dallera P, Bacchini P, Marchetti C, Campobassi A (1991). The Istituto Rizzoli-Beretta experience with osteosarcoma of the jaw. Cancer.

[22] Maor G, Segev Y, Phillip M (1999). Testosterone stimulats insulin-like growth factor-I and insulin-like growth factor-I-receptor gene expression in the mandibular condyle - A model of endochondral ossification. Endocrinology.

[23] Klembara J (2004). Ontogeny of the palatoquadrate and adjacent lateral craneal wall of the endocranium in prehatching Alligator mississippiensis (Archosauria: Crocodylia). J Morphol.

[24] Captier G, Cristol R, Montoya P, Prudhomme M, Godlewski G (2003). Prenatal organization and morphogenesis of the sphenofrontal suture in humans. Cells Tissues Organs.

[25] Eames BF, de la Fuente L, Helms JA (2003). Molecular ontogeny of the skeleton. Birth Defects Res C Embryo Today.

[26] Matsumura G, England MA, Uchiumi T, Kodama G (1994). The fusion of ossification centers in the cartilaginous and membranous parts of the occipital squama in human fetuses. J Anat.

[27] Vanderschueren D, Vandenput L, Boonen S, Lindberg MK, Bouillon R, Ohlsson C (2004). Androgens and bone. Endocr Rev.

[28] Chung UI, Kawaguchi H, Takato T, Nakamura K (2004). Distinct osteogenic mechanism of bones of distinct origins. J Orthop Sci.

[29] Nah HD, Pacifici M, Gerstenfeld LC, Adams SL, Kirsch T (2000). Transient chondrogenic phase in the intramembranous pathway during normal skeletal development. J Bone Miner Res.

